# *Stauntonia hexaphylla* (Lardizabalaceae) leaf methanol extract inhibits osteoclastogenesis and bone resorption activity via proteasome-mediated degradation of c-Fos protein and suppression of NFATc1 expression

**DOI:** 10.1186/s12906-015-0801-6

**Published:** 2015-08-14

**Authors:** Yoon-Hee Cheon, Jong Min Baek, Sun-Hyang Park, Sung-Jun Ahn, Myeung Su Lee, Jaemin Oh, Ju-Young Kim

**Affiliations:** Department of Anatomy, School of Medicine, Wonkwang University, Iksan, Jeonbuk 570-749 Republic of Korea; Center for Metabolic Function Regulation, Wonkwang University, Iksan, Jeonbuk 570-749 Republic of Korea; Division of Rheumatology, Wonkwang University, Iksan, Jeonbuk 570-749 Republic of Korea; Institute for Skeletal Disease, Wonkwang University, Iksan, Jeonbuk 570-749 Republic of Korea; Imaging Science based Lung and Bone Diseases Research Center, Wonkwang University, Iksan, 570-749 Republic of Korea

**Keywords:** *Stauntonia hexaphylla*, Osteoclast, c-Fos, NFATc1, Bone resorption

## Abstract

**Background:**

Natural plants, including common vegetables and fruits, have been recognized as essential sources for drug discovery and the development of new, safe, and economical medicaments. *Stauntonia hexaphylla* (Lardizabalaceae) is widely distributed in Korea, Japan, and China, and is a popular herbal supplement in Korean and Chinese folk medicine owing to its analgesic, sedative, and diuretic properties. However, the exact pharmacological effects of *S. hexaphylla* extract, particularly its effect on osteoclastogenesis, are not known.

**Methods:**

Osteoclast differentiation and function were identified with tartrate-resistant acid phosphatase (TRAP) staining and bone resorption assay, and the underling mechanisms were determined by real-time RT-PCR and western blot analysis.

**Results:**

*S. hexaphylla* was found to inhibit early-stage receptor activator of nuclear factor-κB (NF-κB) ligand (RANKL)-mediated osteoclast differentiation in bone marrow macrophages (BMMs) without cytotoxicity and bone-resorbing activity in mature osteoclasts in a dose-dependent manner. This *S. hexaphylla*-mediated blockade of osteoclastogenesis involved abrogation of the NF-κB, ERK, and c-Src-Btk-PLCγ2 calcium signal pathways. Interestingly, we found that *S. hexaphylla* down-regulated RANKL-associated c-Fos protein induction by suppressing its translation. Furthermore, ectopic overexpression of *c-Fos* and *NFATc1* rescued the inhibition of osteoclast differentiation by *S. hexaphylla*. Furthermore, *S. hexaphylla* inhibited the c-Fos- and NFATc1-regulated expression of genes required for osteoclastogenesis, such as *TRAP*, *OSCAR*, *β3-integrin*, *ATP6v0d2*, and *CtsK.*

**Conclusions:**

These findings suggest that *S. hexaphylla* might be useful for the development of new anti-osteoporosis agents.

## Background

Osteoporosis, characterized by abnormally low bone mineral density and micro-architectural degeneration of bone tissue, is a progressive systemic skeletal disease with various causes, including menopause; advanced age; low calcium and vitamin D intake; and lack of exercise due to long-term bed rest, especially in the elderly population [1–3]. Current anti-osteoporosis drugs such as estrogen, raloxifene, bisphosphonates, and calcitonin have been widely used, and their bone protective mechanisms are based on the inhibition osteoclastic bone resorption. However, these drugs have numerous side effects, causing many patients to discontinue their use [4–7]. Hence, to prevent or reverse osteolytic bone diseases, it is necessary to develop novel agents that have fewer undesirable side effects.

Osteoclasts are specialized, multinucleated, and bone-resorbing cells that form part of the mononuclear phagocyte system [1]. They are formed by the fusion of circulating mononuclear precursor cells of hematopoietic origin. Osteoclast differentiation is critically regulated by macrophage colony-stimulating factor (M-CSF) and receptor activator of nuclear factor-kappa B (NF-κB) ligand (RANKL) [1, 8, 9]. M-CSF stimulates the expression of RANK, the receptor for RANKL, and supports the survival and proliferation of osteoclast precursors [9]. RANKL stimulates differentiation of precursors into osteoclasts by inducing the expression of osteoclast-specific genes, including tartrate-resistant acid phosphatase (*TRAP*), osteoclast-associated receptor (*OSCAR*), and cathepsin K (*CtsK*) [1, 8, 10]. The RANK/RANKL pathway is considered to be an attractive therapeutic target for bone destructive diseases. Although a more detailed mechanism is still expected to be unveiled, the major signaling events have been elucidated: RANK/RANKL binding activates downstream early signaling pathways, including mitogen-activated protein kinase (MAPK) pathways and NF-κB, by recruitment of TNF-receptor associated factor 6 (TRAF6), which regulates transcription factors involved in osteoclast differentiation [11, 12]. Subsequently, RANKL induces the expression of c-Fos and nuclear factor of activated T-cells, cytoplasmic 1 (NFATc1), key transcription factors for osteoclastogenesis [13–15]. NFATc1 is a master regulator of terminal osteoclastogenesis, and autoamplification of NFATc1 depends on activator protein (AP)-1 complex containing c-Fos and NF-κB [15]. In addition, the RANKL/RANK signaling pathway activates Ca^2+^ signaling through the activation of phospholipase Cγ (PLCγ) [16, 17]. PLCγ regulates protein kinase C (PKC) activation, intracellular Ca^2+^ levels, and NFATc1 expression in hematopoietic systems [16, 18]. During osteoclastogenesis, increased Ca^2+^ levels induce NFATc1 dephosphorylation and NFATc1 translocation into the nucleus. Calcineurin inhibitors, such as FK506 and cyclosporine A, as well as the Ca^2+^ chelator BAPTA-AM, potently suppress RANKL-induced osteoclastogenesis through inhibition of NFATc1 nuclear translocation [19].

Recently, many plants and their extracts have been recognized as useful sources for the prevention and treatment of bone-related disorders. *Stauntonia hexaphylla* (Lardizabalaceae) is widely distributed in thickets in lowlands and foothills in warmer regions of Korea, Japan, and China. *S. hexaphylla* has been used in Chinese folk medicine as analgesics, sedatives, and diuretics [20]. As part of a search for bioactive compounds, methanol extract from the aerial part of *S. hexaphylla* was found to exhibit significant cytotoxic activity and anti-cancer effects on HCT116 human colon cancer cells [21]. However, the exactly pharmacological effect of *S. hexaphylla* remains unknown; in particular, the effect of *S. hexaphylla* on osteoclast differentiation and pathological bone destruction has not yet been well defined. In this study, we have investigated the effects of *S. hexaphylla* on osteoclast differentiation and function, and its possible mechanism of action.

## Methods

### Reagents and antibodies

Methanol extract from the *S. hexaphylla* leaf was purchased from the Korean Plant Extract Bank (Daejeon, Korea). Recombinant soluble human M-CSF and RANKL were obtained from PeproTech EC Ltd. (London, UK). Monoclonal β-actin antibody was obtained from Sigma (St. Louis, MO, USA). Anti-Akt, anti-phospho-Akt, anti-p38, anti-phospho-p38, anti-JNK, anti-phospho-JNK, anti-phospho-IκB, and anti-phospho-PLCγ2 were purchased from Cell signaling Technology Inc. (Beverly, MA, USA). Anti-c-Fos, anti-NFATc1, anti-IκB, and anti-PLCγ2 were purchased from Santa Cruz Biotechnology (Santa Cruz, CA, USA). Fetal bovine serum (FBS), α-minimum essential medium (α-MEM), and penicillin/streptomycin were purchased from Gibco BRL (Grand Island, NY, USA). All other chemicals were of analytical grade or complied with the standards required for cell culture experiments. Cyclohexamide (CHX), MG132, and Ac-Leu-Leu-nle-h (ALLN) were obtained from Calbiochem (San Diego, CA, USA).

### Mice and *in vitro* osteoclastogenesis assay

BMMs isolation was performed in accordance with the guidelines for animal experimentation of the Institutional Animal Care and Use Committee of Wonkwang University (WKU15-48, committee member: Sung Yeon Kim, Jungkee Kwon, Hong Geun Oh, Hong-Seob So, Okjin Kim, Chun-Soo Ko). Five-week-old male ICR mice were purchased from Damul Science (Daejeon, Korea). The mice were housed in cages in a controlled temperature (22–24 °C) and humidity (55–60 %) room with a 12 h light/dark cycle. Bone marrow cells (BMCs) were obtained from 5-week-old male ICR mice by flushing the tibias and femurs with α-MEM supplemented with 10 % FBS, penicillin (100 U/mL), and streptomycin (100 μg/mL). To obtained bone marrow macrophages (BMMs) for osteoclast differentiation of primary BMCs, the cells were cultured in α-MEM supplemented with 10 % FBS and M-CSF (10 ng/mL) for 1 day on culture dishes. Non-adherent cells were transferred to 10 cm petri dishes and further cultured in the presence of M-CSF (30 ng/mL) for 3 days. Adherent cells were used as BMMs or as osteoclast precursors, after the non-adherent cells were removed. To generate osteoclasts from the BMMs culture system, cells were cultured with M-CSF (30 ng/mL) and RANKL (100 ng/mL) in the presence or absence of *S. hexaphylla* on 48-well plates, placed in an incubator set to 37 and 5 % CO_2,_ and allowed to incubate for 3 days. The cells were fixed in 3.7 % formalin for 10 min, permeabilized with 0.1 % Triton X-100, and then stained with TRAP solution. TRAP-positive multinucleated cells (MNCs) with more than three nuclei were counted as osteoclasts.

### PCR primers and quantitative real-time RT-PCR

Oligonucleotides used in this experiment were commercially synthesized by Bioneer Co. (Daejeon, Korea). Real-time RT-PCR analysis was performed using an ExicyclerTM 96 Real-Time Quantitative Thermal Block (Bioneer Co.). The real-time RT-PCR reactions were conducted with initial denaturation for 10 min at 95 °C, followed by 40 cycles of denaturation for 1 min at 95 °C, annealing for 30 s at 60 °C, and extension for 1 min at 72 °C. The expression of target molecules was analyzed using SYBR Green-based real-time RT-PCR, and normalized to the expression of *GAPDH*. The primer sets used in the quantitative real-time RT-PCR are shown in Table 1.

**Table 1:** The oligonucleotides used as primers

Gene	Forward primer (5'-3')	Reverse primer (5'-3')
*c-Fos*	GGTGAAGACCGTGTCAGGAG	TATTCCGTTCCCTTCGGATT
*NFATc1*	GAGTACACCTTCCAGCACCTT	TATGATGTCGGGGAAAGAGA
*TRAP*	ACTTCCCCAGCCCTTACTACCG	TCAGCACATAGCCCACACCG
*OSCAR*	GGAATGGTCCTCATCTCCTT	TCCAGGCAGTCTCTTCAGTTT
*CtsK*	CCAGTGGGAGCTATGGAAGA	CTCCAGGTTATGGGCAGAGA
*β3-integrin*	GGAGTGGCTGATCCAGATGT	TCTGACCATCTTCCCTGTCC
*ATP6v0d2*	GACCCTGTGGCACTTTTTGT	GTGTTTGAGCTTGGGGAGAA
*GAPDH*	TCAAGAAGGTGGTGAAGCAG	AGTGGGAGTTGCTGTTGAAGT

### Cytotoxicity assay

Cell proliferation was measured using the XTT assay kit (Sigma). The BMMs (1 × 10^4^/well) were seeded in 96-well plates with various concentrations of *S. hexaphylla* and cultured for 3 days in the presence of M-CSF (30 ng/mL). For the last 4 h of culture, 50 μL of XTT solution was added, and the plate was read at 450 nm with an ELISA reader (Molecular Devices, CA, USA).

### Western blot analysis

After the specified treatment, cells were washed twice with cold-PBS and lysed with lysis buffer containing 50 mM Tris–HCl, 150 mM NaCl, 5 mM EDTA, 1 % Triton X-100, 1 mM sodium fluoride, 1 mM sodium vanadate, 1 % deoxycholate, and protease inhibitors. The protein concentration was determined using a Bio-Rad DC protein assay kit (Bio-Rad Laboratories., Hercules, CA, USA). The same amount of protein (15–30 μg) was separated using 10 % SDS-polyacrylamide gel and transferred to a polyvinylidene difluoride (PVDF, Millipore, Bedford, MA, USA) membrane for 90 min at 25 V on an XCell II blot module (Invitrogen, Carlsbad, CA, USA). The PVDF membranes were placed in 5 % nonfat milk in tris-buffered saline with 0.1 % Tween 20 (TBST) for 1 h, washed, and incubated with the primary antibodies for 2 h at room temperature. The membranes were washed in TBST, and incubated for 1 h with various HRP-conjugated donkey anti-rabbit, sheep anti-mouse IgG secondary antibodies. The immunoreactive signals were detected using the Western Chemiluminescent HRP substrate kit (Millipore, Billerica, USA).

### Bone resorption assay

Primary osteoblast cells (1 × 10^6^ cells) and BMCs (1 × 10^7^ cells) were cultured on collagen-gel-coated culture dishes for 7 days in the presence of 10^−8^ M 1,25-dihydroxyvitamin D3 (Sigma) and 10^−6^ M prostaglandin E_2_ (PGE_2_) (Sigma). The co-cultured osteoclasts were detached by 0.1 % collagenase treatment at 37 °C for 10 min and then placed on dentine slices or hydroxyapatite coated plates (Corning, NY, USA) with or without *S. hexaphylla*. After 24 h, the cells were removed, and the total resorption pits were observed under a microscope and then quantified using Image-Pro Plus version 4.0 (Media Cybernetics, Silver Spring, MD, USA).

### Retrovirus preparation and infection

Plat-E retroviral packaging cells were transfected by pMX-IRES-EGFP, pMX-c-Fos-IRES-EGFP, and pMX-CA-NFATc1-IRES-EGFP using X-tremeGENE 9 (Roche, Nutley, NJ, USA) according to the manufacturer’s protocol. The culture supernatant of the retrovirus-producing cells was collected for retroviral infection on BMMs that were cultured with BMCs in M-CSF (30 ng/mL) for 2 days. The BMMs were incubated with the retrovirus soup produced by Plat-E cells together with polybrene (10 μg/mL) and M-CSF (10 ng/mL) for 6 h. Infected BMMs were further cultured in the presence of M-CSF (30 ng/mL) and RANKL (100 ng/mL) with or without *S. hexaphylla* for 3 days. Osteoclast formation was detected by TRAP staining.

### c-Fos stability

The BMMs were incubated with c-Fos retro-virus soup produced by Plat-E cells together with polybrene (10 μg/mL) and M-CSF (10 ng/mL) for 6 h. Infected BMMs were pretreated with or without *S. hexaphylla* in the presence of M-CSF for 24 h, and then stimulated with RANKL. After 20 h, 2 μg/mL CHX, 5 μM MG132, and 20 μM ALLN were added to the cultures for 4 h before harvest.

### Statistical analysis

Experiments were conducted at least three times, and the data are expressed as the mean ± standard deviation (SD). All statistical analyses were conducted using the Statistical Package for the Social Sciences Software (SPSS; Korean version 14.0). Most of the statistical differences were analyzed using one-way ANOVA followed by Tukey’s post hoc test. *P*-values of less than 0.05 were considered statistically significant.

## Results

### *S. hexaphylla* suppresses RANKL-induced osteoclast differentiation in BMMs and bone resorption by co-cultured mature osteoclast

**Fig. 1:**
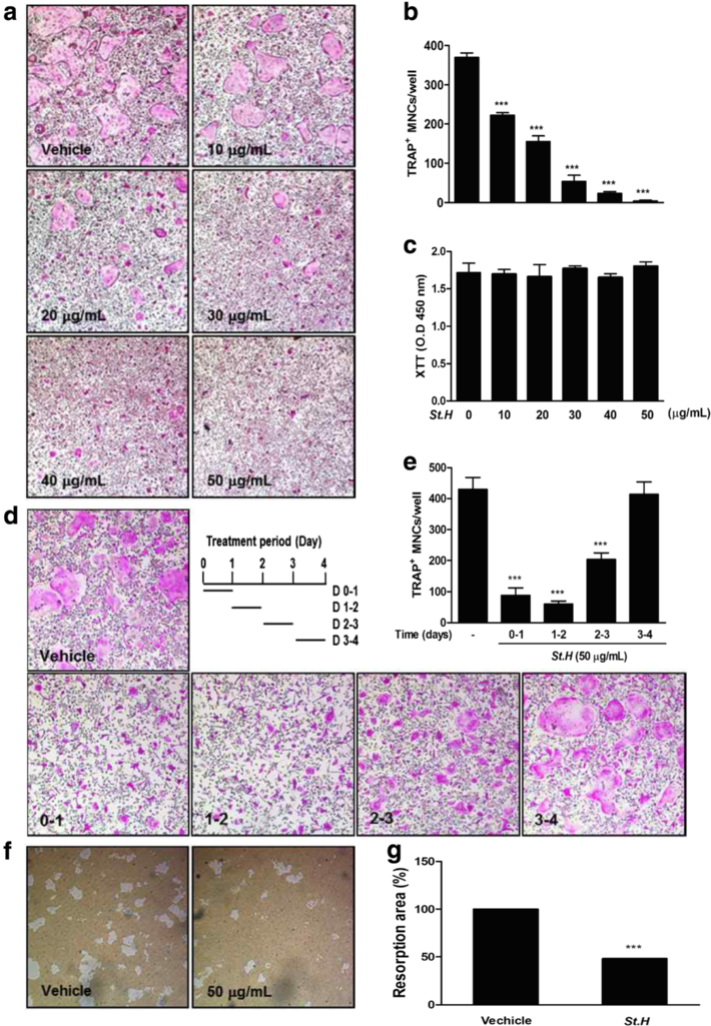
*S. hexaphylla* suppresses RANKL-induced osteoclast differentiation in BMMs and bone resorption by co-cultured mature osteoclasts. **a** BMMs were cultured with M-CSF (30 ng/mL) and RANKL (100 ng/mL) for 3 days in the presence or absence of *S. hexaphylla*. **b** After culturing, cells were fixed with 3.7 % formalin in PBS, permeabilized with 0.1 % Triton X-100 in PBS, and stained with TRAP staining solution; TRAP-positive MNCs were then counted. **c** BMMs were cultured for 3 days with the indicated doses of *S. hexaphylla* in the presence of M-CSF (30 ng/mL). Cell viability was analyzed using an XTT assay. **d** BMMs were cultured as in (**a**), except that the cells were treated with *S. hexaphylla* on the indicated days. **e** After culturing, cells were fixed, and the number of TRAP-positive MNCs was counted. **f** pOBs and BMCs were co-cultured on a collagen-matrix-coated plate for 6 days, and the flourishing osteoclasts were harvested. Mature osteoclasts were seeded on hydroxyapatite-coated plates with or without *S. hexaphylla* for 24 h. Attached cells on the plates were removed and photographed under a light microscope. Pit areas were quantified using Image J (**g**), and bone resorption numbers were counted using a light microscope. ^***^ *P* < 0.001; versus vehicle (DMSO)

To identify the efficacy of *S. hexaphylla* on RANKL-induced osteoclastogenesis, BMMs were cultured in the presence of RANKL and M-CSF treated with or without various concentrations of *S. hexaphylla*. However, RANKL differentiated the BMMs of the control into TRAP-positive MNCs, and *S. hexaphylla* dose-dependently decreased the formation of TRAP- positive MNCs (Fig. 1a and b). Next, we evaluated the XTT assay to determine whether the inhibitory effect of *S. hexaphylla* on osteoclastogenesis was due to reduced viability or proliferation of the osteoclast precursor cells. *S. hexaphylla* had no cytotoxic effects, did not affect cell proliferation, and inhibited osteoclast differentiation (Fig. 1c). To determine the time course of the inhibitory effect of *S. hexaphylla* on osteoclast differentiation, BMMs were cultured by treatment with *S. hexaphylla* (50 μg/mL) at four different time points with RANKL and M-CSF. The cells incubated for 24 h in an *S. hexaphylla*-containing medium, and were then transferred to an *S. hexaphylla*-free medium. As shown in Fig. 1d*,* the time frames in which *S. hexaphylla* most effectively suppressed osteoclast formation were days 0–1 and 1–2. However, its inhibitory effect was slightly weaker on days 2–3 and 3–4 (Fig. 1d and e). These finding suggest that *S. hexaphylla* exerts an inhibitory effect on early differentiation. We next examined whether *S. hexaphylla* can reduce the function of mature osteoclasts. Mature osteoclasts were cultured on hydroxyapatite-coated plates in the presence of *S. hexaphylla*. The addition of *S. hexaphylla* clearly inhibited bone resorption activity relative to the DMSO control (Fig. 1f and g).

### *S. hexaphylla* suppresses RANKL-induced ERK, NF-κB, and Ca^2+^ signaling pathways

**Fig. 2:**
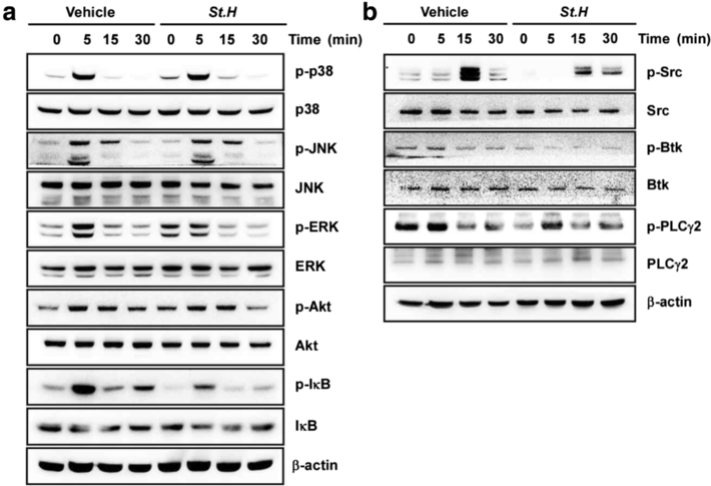
*S. hexaphylla* suppresses RANKL-induced ERK, NF-κB, and Ca^2+^ signaling pathways. BMMs were starved with serum-free α-MEM media for 3 h. BMMs were pretreated with *S. hexaphylla* for 1 h and then stimulated with RANKL for the indicated times. BMMs were pretreated with or without *S. hexaphylla* for 1 h and then stimulated with RANKL (100 ng/mL) for the indicated time. The cell lysates were analyzed by western blotting with antibodies. β-actin was used as the internal control

RANKL binding to RANK results in the recruitment of multiple downstream signaling pathways such as Akt, p38, JNK, ERK, and NF-κB. We examined the effect of *S. hexaphylla* on RANKL-induced early signaling. As shown in Fig. 2a, RANKL-induced phosphorylation of p38, JNK, and Akt was not affected by *S. hexaphylla* treatment, but *S. hexaphylla* inhibited the phosphorylation of ERK and degradation of IκB. We next examined whether *S. hexaphylla* alters the RANKL-PLCγ2-Ca^2+^-related signal pathway in which Ca^2+^ oscillation is followed by promotion of NFATc1. *S. hexaphylla* completely blocked RANKL-mediated c-Src, Btk, and PLCγ2 phosphorylation (Fig. 2a and b).

### *S. hexaphylla* downregulates RANKL-induced expression of NFATc1 and c-Fos stability

**Fig. 3:**
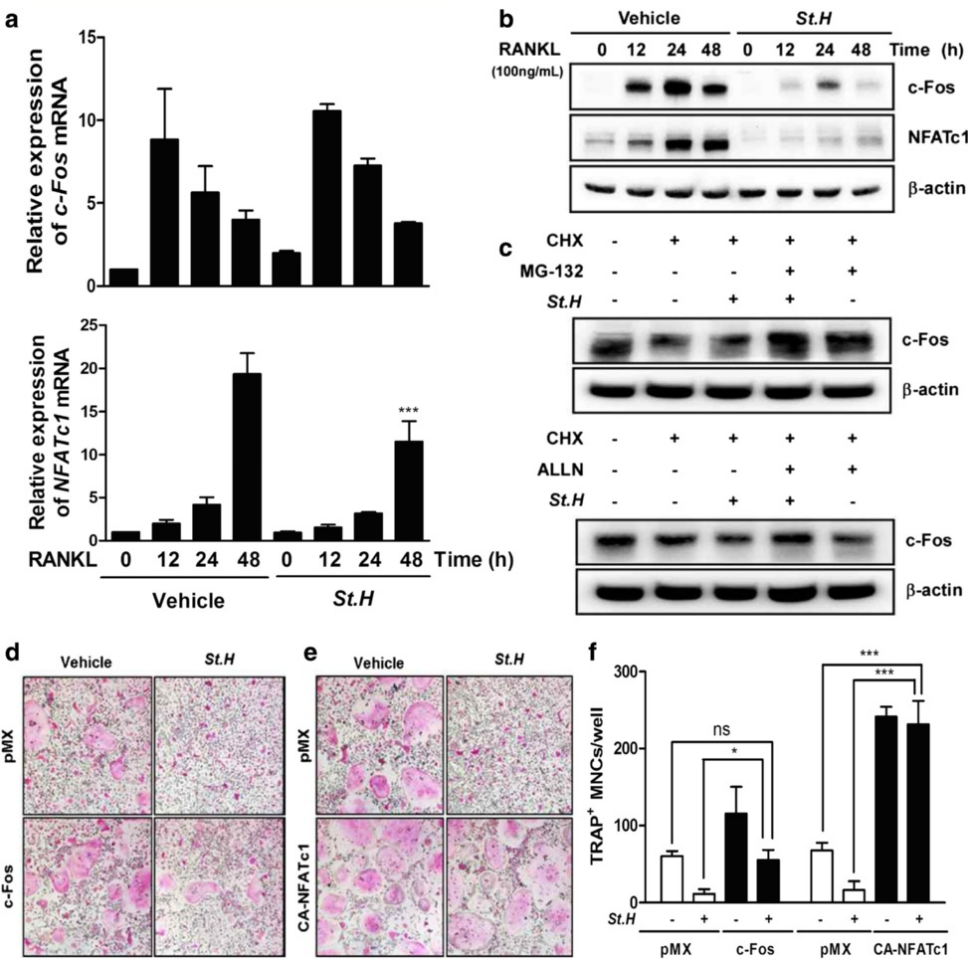
*S. hexaphylla* downregulates RANKL-induced expression of NFATc1 and c-Fos stability. BMMs were pretreated with *S. hexaphylla* for 1 h and then stimulated with RANKL (100 ng/mL) for the indicated times. Total RNA or protein of c-Fos and NFATc1 were obtained at the indicated time points, respectively. **a** mRNA expression of *c-Fos* and *NFATc1* was analyzed by real-time RT-PCR. **b** Western blot analysis was performed with the indicated antibodies. β-actin was used as an internal control. **c** BMMs were infected with pMX-c-Fos-IRES-EGFP (*c-Fos*). Infected BMMs were pretreated with or without *S. hexaphylla* in the presence of M-CSF (30 ng/mL) for 24 h and then stimulated with RANKL (100 ng/mL). After 20 h, 2 μg/mL CHX, 5 μM MG132, and 20 μM ALLN were added to the cultures for 4 h before harvest. Western blot analysis was then performed on the cells. **d** BMMs were infected with pMX-IRES-EGFP (control vector), pMX-c-Fos-IRES-EGFP (*c-Fos*), or (**e**) pMX-CA-NFATc1-IRES-EGFP (CA-*NFATc1*). After infection, the cells were cultured in the presence of M-CSF and RANKL with or without *S. hexaphylla*. **f** TRAP-positive MNCs were counted. ^***^ *P* < 0.001; versus vehicle (DMSO). ns: not significant; ^***^ *P* < 0.001; ^*^ *P* < 0.05 versus vehicle (DMSO)

c-Fos and NFATc1 are key transcription factors in RANKL-induced osteoclast differentiation, and they regulate expression of the genes involved in osteoclast differentiation and function such as *TRAP, OSCAR,* and *CtsK.* Therefore, we investigated *c-Fos* and *NFATc1* mRNA expression. As previously reported, c-Fos and NFATc1 expression was upregulated in BMMs by RANKL stimulation. *S. hexaphylla* decreased the increased expression of the c-Fos protein by RANKL without any marked changes in *c-Fos* mRNA expression (Fig. 3a and b). Next, we examined whether *S. hexaphylla* promotes c-Fos protein degradation. After 48 h post-transfection with c-Fos, the cells treated with CHX, an inhibitor of protein synthesis, decreased c-Fos protein expression compared to control (Fig. 3c); additional *S. hexaphylla* treatment accelerated this decrease in c-Fos protein levels; and the effect of *S. hexaphylla* was reversed by co-treatment with selective proteasome inhibitors, namely, MG132 or ALLN. These results suggest that proteasome-mediated degradation is related to the *S. hexaphylla*-induced reduction in c-Fos protein (Fig. 3c). Next, we examined whether forced expression of c-Fos and NFATc1 was sufficient to reverse the inhibitory effect of *S. hexaphylla* on osteoclast differentiation. The ectopic expression of c-Fos and NFATc1 fully reversed the inhibitory effect of *S. hexaphylla* on RANKL-induced osteoclast differentiation (Fig. 3d-f). These results indicate that the effect of *S. hexaphylla* may involve the inhibition of c-Fos and NFATc1 during RANKL-mediated osteoclastogenesis.

### *S. hexaphylla* inhibits RANKL-induced mRNA expression of *OSCAR, TRAP, ATP6v0d2*, *β3-integrin*, and *CtsK*

**Fig. 4:**
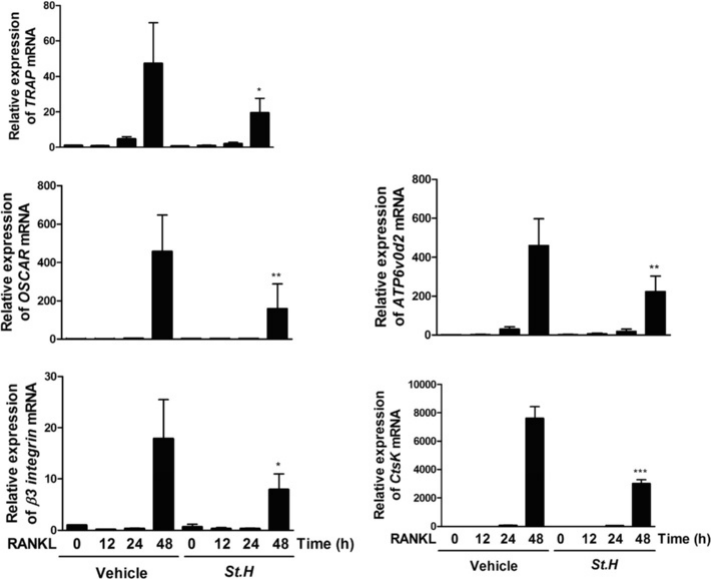
*S. hexaphylla* inhibits RANKL-induced mRNA expression of *OSCAR, TRAP, ATP6v0d2, β3*-*integrin,* and *CtsK.* BMMs were pretreated with *S. hexaphylla* for 1 h and then stimulated with RANKL (100 ng/mL) for the indicated times. Total RNA was obtained at the indicated time points, respectively. The mRNA expression of *OSCAR, TRAP, ATP6v0d2, β3*-*integrin,* and *CtsK* was analyzed by real-time RT-PCR. ns: not significant; ^***^ *P* < 0.001; ^**^ *P* < 0.01; ^*^ *P* < 0.05 versus vehicle (DMSO). ^***^ *P* < 0.001; ^**^ *P* < 0.01; ^*^ *P* < 0.05 versus vehicle (DMSO)

NFATc1 regulates the expression of *OSCAR, TRAP, ATP6vod2, β3-integrin,* and *CtsK* during RANKL-induced osteoclastogenesis. We determined whether *S. hexaphylla* regulates the expression of *OSCAR, TRAP, ATP6vod2, β3-integrin,* and *CtsK. S. hexaphylla* down-regulated the expression of OSCAR and TRAP, which are genes related to osteoclast formation. *S. hexaphylla* also decreased the expression of *ATP6vod2* and *β3-integrin,* which affect cell-to-cell migration or fusion. The expression of *CtsK* and its associated bone-resorbing activity was inhibited by *S. hexaphylla* (Fig. 4), suggesting that the inhibitory effect of *S. hexaphylla* on RANKL-mediated NFATc1 expression is followed by the down-regulation of osteoclastogenic marker genes.

## Discussion

There is a growing interest in the efficacy of natural plant products for the prevention and treatment of bone-related disorders such as osteoporosis and rheumatoid arthritis. In this study, we have shown that *S. hexaphylla* effectively inhibits RANKL-induced osteoclast differentiation at the early stages without any cytotoxicity or bone-resorbing activity by mature osteoclasts (Fig. 1).

RANK/RANKL interaction forms a fundamental cytokine system that is capable of influencing all aspects of osteoclast differentiation, function, and (indirectly) the complete bone regulatory system [10, 12]. The connection between RANK and RANKL induces recruitment and activation of TRAF6, which leads to multiple downstream cascades. The signals are composed of six known signal pathways: ERK, p38, JNK, NF-κB, Src, and Akt. We found that by suppressing RANKL-mediated ERK and NF-κB signaling, *S. hexaphylla* inhibited osteoclast differentiation (Fig. 2a). In previous studies, compared to WT mice, ERK1 deficiency reduced the number of osteoclast progenitors and the formation of TRAP-positive cells on the trabecular surface [22]. In addition, NF-κB is an important signal mediator of inflammatory and immune reactions, and is a major transcription factor for RANKL-activated osteoclastogenesis [23]. IκB is bound to NF-κB, which prevents it from translocating to the nucleus, and phosphorylation by IKK separates the two proteins. Subsequent ubiquitination and proteasomal degradation of IκB allows NF-κB to translocate into the nucleus and stimulate the transcription of the target gene [24]. NF-κB p50/p52 double knockout mice exhibit osteoclastogenesis defects and severe osteopetrosis [25, 26]. *S. hexaphylla* also induces osteoclastogenesis through the c-Src-Btk-PLCγ2 signaling pathway (Fig. 2b). c-Src deficiency produces an osteopetrotic skeletal phenotype and affects the bone-resorbing activity of mature osteoclasts [27, 28]. PLCγ activates Ca^2+^ signaling and NFATc1 in immune cells. PLCγ2 deficiency in mouse cells lowers the expression of NFATc1 RANKL-stimulation relative to wild mice [29].

Under normal conditions, the RANKL-RANK axis appears to be essential for osteoclastogenesis, and costimulatory immunoreceptors lead to robust induction of c-Fos and NFATc1, which are the necessary and sufficient transcription factors for osteoclast differentiation. The crucial role of c-Fos in osteoclastogenesis was demonstrated by *in vivo* experiments using genetically modified mice. c-Fos-deficient mice exhibit a severe osteoporotic phenotype due to the failure of osteoclast differentiation [30]. NFATc1-deficient embryonic stem cells are unable to differentiate into osteoclasts in response to RANKL, and the forced expression of NFATc1 leads to the formation of osteoclasts from BMMs in the absence of RANKL [15]. In our study, *S. hexaphylla* suppressed RANKL-induced NFATc1 mRNA and protein expression. By contrast, c-Fos protein, but not mRNA, expression was diminished (Fig. 3a and b). This suggests that *S. hexaphylla*-mediated inhibition of NFATc1 expression is not involved in c-Fos transcriptional activity, and instead results from the inhibition of c-Fos translational activity. Moreover, the protein stability assay showed that the reduction in c-Fos protein contributed to the suppression of c-Fos translation via proteasome-dependent degradation (Fig. 3c). Furthermore, the forced expression of *c-Fos* or CA-*NFATc1* rescued the *S. hexaphylla*-induced inhibition of osteoclast differentiation, which suggests that down-regulation of c-Fos is responsible for the inhibitory effects of *S. hexaphylla* (Fig. 3d-f).

NFATc1 has a crucial role in the regulation of osteoclastogenic marker genes during RANKL-mediated osteoclast differentiation, and it gradually induces the expression of osteoclast-specific genes, including *OSCAR*, *TRAP*, *ATP6v0d2*, *β3-integrin*, and *CtsK* [1, 8, 9]. The present data suggest that *S. hexaphylla* suppressed the induction of *OSCAR, TRAP, ATP6v0d2*, *β3-integrin*, and *CtsK* (Fig. 4). Several proteolytic enzymes, including OSCAR, TRAP, *ATP6v0d2,* and *CtsK*, have been shown to play important roles in degrading the organic bone matrix, and of these proteolytic enzymes have the highest levels in osteoclasts. Matrix-degrading enzymes are also known to be collagen-degrading enzymes, and can directly degrade collagen in hard tissues that have been demineralized. In addition, *ανβ3-integrin* is known to play a role in the regulation of cell migration and the maintenance of the sealing zone required for effective osteoclastic bone resorption. This indicates that *S. hexaphylla* extract inhibited bone resorption by disrupting the induction of cytoskeletal organization, leading to the formation of a ruffled border and the adhesion of osteoclasts to the bone matrix.

## Conclusions

In conclusion, this is the first study to report that *S. hexaphylla* efficiently suppresses RANKL-induced osteoclast differentiation and bone-resorbing activity *in vitro. S. hexaphylla* inhibits osteoclastogenesis via proteasome-mediated degradation of c-Fos protein and suppression of NFATc1 induction. These results suggest that *S. hexaphylla* has potential as a natural herbal therapy for diseases associated with bone loss.
